# Gastruloids — a minimalistic model to study complex developmental metabolism

**DOI:** 10.1042/ETLS20230082

**Published:** 2023-12-18

**Authors:** Chaitanya Dingare, Ben Steventon

**Affiliations:** Department of Genetics, University of Cambridge, Downing Site, Cambridge CB2 3EH, U.K.

**Keywords:** gastrulation, gastruloids, glycolysis, metabolism

## Abstract

Metabolic networks are well placed to orchestrate the coordination of multiple cellular processes associated with embryonic development such as cell growth, proliferation, differentiation and cell movement. Here, we discuss the advantages that gastruloids, aggregates of mammalian embryonic stem cells that self-assemble a rudimentary body plan, have for uncovering the instructive role of metabolic pathways play in directing developmental processes. We emphasise the importance of using such reductionist systems to link specific pathways to defined events of early mammalian development and their utility for obtaining enough material for metabolomic studies. Finally, we review the ways in which the basic gastruloid protocol can be adapted to obtain specific models of embryonic cell types, tissues and regions. Together, we propose that gastruloids are an ideal system to rapidly uncover new mechanistic links between developmental signalling pathways and metabolic networks, which can then inform precise *in vivo* studies to confirm their function in the embryo.

## Introduction

Studies focusing on the metabolic control of embryonic development are decades old, involving a wide variety of organism from planarians, hydroids to vertebrates such as chickens [1–4]. Nonetheless, the focus has always been on understanding the genetic and molecular basis of embryonic development. This tug-of-war between genetics and metabolic studies is decades old. The apparent conflict between the two approaches was clear from Charles Manning Child's experiments and his contemporary Thomas Morgan. Child proposed the theory of metabolic gradients which act as a mediator between the environment and the organism and its impact the embryonic development [1,5,6]. Morgan, as a *Drosophila* geneticist, used genetics to explain a phenotype [7,8]. The criticisms Child faced were the use of the chemical toxins and the lack of a simple explanation of the phenotypes he had observed [1,9]. In contrast, the field of developmental genetics has offered a tool to probe the role of gene function in embryonic development and molecular biology has given the tools to follow key processes such as embryonic induction with tight temporal precision [10]. A new picture is emerging, however, where metabolites are increasingly being shown to act as signals themselves, directing cellular behaviours in both physiology and development [11]. As recent studies have focussed primarily on the connection between central carbon metabolism and developmental signalling pathways, this will be reflected in the focus of this review article.

At the time of Child and Morgan, the field lacked an inclusive conceptual framework that could bring their work under a single umbrella to explain dynamic biological processes such as embryonic development. However, with modern technology and conceptual frameworks that bridge multiple scales of biological organisation, we are now in a new era of integration in approaches to developmental biology [12]. Similar technological advances in the field of metabolism have given rise to the separate field of metabolomics. With such all-round progress, we are better placed to revive the field of developmental metabolism. As Jane Oppenheimer has pointed out (see quote), developmental biology has repeatedly integrated biological approaches through successive waves of anatomy, cell biology, molecular biology and genetics. Here, she comments on how there has been a progression through the study of whole embryos by the likes of Aristotle, Fabricius, Harvey, Malpighi and Wolff, in terms of their separate layers by Wolff, Pander, von Baer, His, Haeckel and Spemann, and then in relation to their cells (Roux, Driesch, Spemann, Harrison and others) and their components (O. Hertwig, Boveri, E. B. Wilson and Conklin). We are now ready to begin a phase of examining the complex relations of metabolism, signalling and molecular epigenetics, and are poised to re-integrate metabolism into this multi-scale framework:

The integrative powers of the embryo, at all of its levels, are however so pervasive that they never permit themselves to be overlooked by those who avail themselves the privilege of looking at the embryo at all. The result has been that when each of the practices just enumerated became fashionable, the previous one was never outmoded; and when, at each stage of its development, embryology has added a new dimension to its studies, it has never wholly discarded the old ones. [13]

Embryonic development involves cellular behaviours such as migration, proliferation and cell growth. It is of utmost importance to co-ordinate all these processes to generate well-proportioned tissues and organs. Cell migration, proliferation and growth are energy expensive events and how their energetic needs are fulfilled has been largely unexplored. Metabolism, as mentioned by Child, acts as a mediator in interpreting environmental cues [6]. There is a vast diversity of organisms which grow, reproduce and flourish in different environmental conditions each having a unique type of food supply. Moreover, each organism has a unique energetic demand that must be fulfilled to support its own physiological processes. Any changes in the environment might further impact embryonic development. Therefore, it is very important to understand how nutritional supply and metabolic needs shape embryonic development and evolution. This is of particular interest to understand, not just the fundamental aspect of metabolic control of embryonic development, but how changes in any of the environmental factors illicit evolutionary changes as organisms adapt to new environments [14].

Unlike externally developing animals, mammalian embryos are exposed to very controlled external environment due to their *in utero* development. Their nutritional demands are fulfilled by the mother and nutrients are passed through the placenta, an extra-embryonic tissue that connects the maternal tissue to the embryo. Such controlled conditions are important for mammals to achieve successful development as the litter size is small. They also offer a very little room for any changes in the physiology of the mother to be accommodated. For example, higher blood glucose during pregnancy, i.e. gestational diabetes leads to developmental abnormalities, and they arise as early as gastrulation when the three germ layers are formed and organised along the three body axes [15,16]. To understand the aetiology of such developmental disorders, it is important to investigate how different nutrients play an important role during mammalian development. This would help to determine the extent to which effects of maternal nutrition play a direct role on embryonic tissues, in the isolation of the additional impact it might have on extra-embryonic tissues. Despite the need to study the role of metabolism and nutrient uptake in mammalian embryos, they are not readily amenable to any metabolic manipulations due to their *in utero* development. Extra-embryonic tissues which act a gatekeeper for nutrients and gases to pass on to the embryo, an exchange that must be fully understood to gain a complete understanding of the metabolic control of early development [17–19].

In the mouse embryo, gastrulation occurs prior to nutrient and gas exchange between the mother and the fetus, a process that begins with chorioallantoic branching and has been shown to be associated with a global re-wiring of embryo metabolism [20]. This review focuses on how the gastrulation stage embryo takes up and metabolises nutrients from the uterine environment prior to this stage. A technical issue associated with studying the stages of mouse development relates to the number of samples required to perform any system-level analysis of metabolites is limited due to tissue availability. To understand the role of metabolism in embryonic development, we need a system that can be produced in large numbers, is amenable to metabolic perturbations and exhibits a reduced biological complexity. One such system is gastruloids, i.e. aggregates of the embryonic stem cells which fits in well with the requirements one has, to study the role of metabolism in mammalian embryogenesis.

## What are gastruloids?

Gastruloids are aggregates of embryonic stem cells which form all the three germ layers and self-organise gene expression patterns reminiscent of the primordia for the three body axes *in vitro*. Subsequently, they undergo axial elongation along the anterior–posterior (AP) axis [21–23]. Mouse gastruloids are made from the naïve pluripotent stem cells and the success of symmetry breaking and axial elongation depends on the stage of pluripotency of the cells used to make aggregates [24]. This particular property makes it easier to grow cells in a more controlled manner to reduce the variability which is essential in metabolic studies. Upon aggregation, a Wnt pathway agonist is used to induce mesodermal fate [22]. These mesodermal cells later become localised to one pole, marking the posterior pole of the gastruloids, and thus breaking the symmetry [23]. Human gastruloids are made in a similar manner except the pluripotent state of human cells at the beginning corresponds to the more advanced state of pluripotency in mouse cells [25]. Overall, gastruloids represent a minimalistic model of patterning and morphogenesis that happen *in vivo*.

## Why gastruloids?

**Figure 1.**
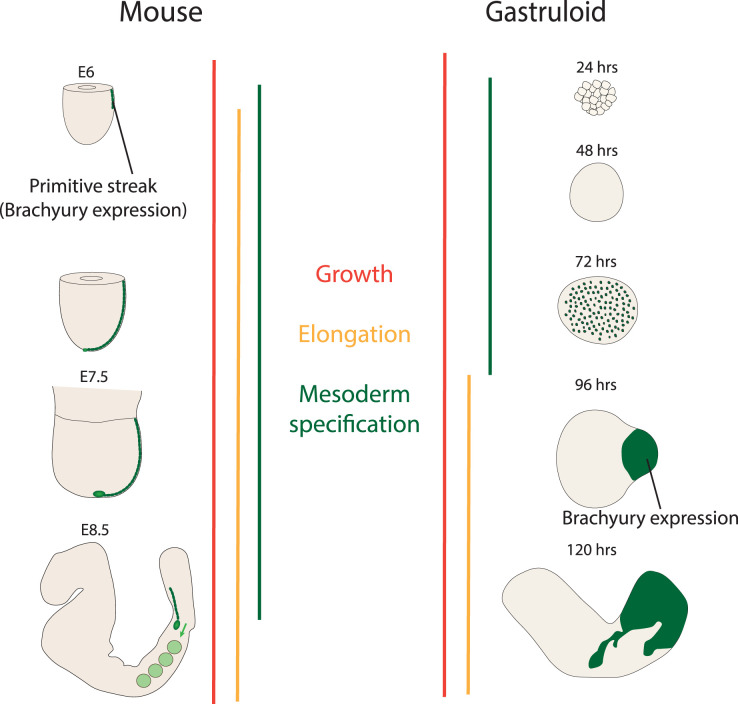
Comparison of developmental processes in mouse embryos and gastruloids. (Left) Mouse embryos undergo substantial amounts of growth and proliferation concomitant with mesoderm specification as cells ingress through the primitive streak (green) and the elongation of the anterior–posterior body axis. (Right) Stages of the mouse gastruloid protocol can be related to equivalent stages of mouse development through comparative RNAseq [21]. Mesoderm specification can be temporally separated from gastruloid elongation allowing for the temporal control over metabolic inhibition experiments to study each process.

Gastruloids, being a minimalistic model, exhibit only a subset of cell behaviours and patterning events that happen *in vivo*. This property makes it easier to address previously unappreciated roles of metabolites during germ layer specification and body axis elongation. In the mouse embryo, mesodermal cells are specified, undergo an epithelial–mesenchymal transition (EMT), ingress through the primitive streak, migrate under the epiblast and become patterned along the prospective dorsal–ventral axis [26] (Figure 1). The combination of patterning and morphogenetic events is energy demanding and requires the integration of multiple developmental signalling pathways making this process ideal for the study of metabolism in development [26–28]. However, uncoupling these events in the context of *in vivo* studies is a major challenge for the field and can be supported by work *in vitro* where specific developmental processes can be isolated to uncover novel connections between metabolism and developmental cell biology. In many ways, this is continuing an experimental embryology approach, where cut-and-paste biology has enabled the elucidation of the molecular mechanisms that underpin morphogenesis and patterning [12,29].

In gastruloids, mesodermal cells are specified on one day and the axial elongation happens on the next day of the gastruloid development, thus temporally separating patterning and morphogenetic events [23] (Figure 1). This enables experiments that can ascribe a role of a particular metabolic pathway to mesodermal patterning and/or axial elongation through the application of perturbations in defined temporal windows. Such hurdles are apparent in mouse mutants targeting the glucose transporters, hexokinase II or glucose phosphate isomerase where the entire embryo looks disorganised having a complex phenotype at both patterning and morphogenesis levels [30–33]. Indeed, the mouse genetics have enabled us to create conditional knock-out alleles that would help address this issue. Nevertheless, generating such alleles is cumbersome, and there is a risk of not having a developmental function in a chosen time window or in a cell type under study. Therefore, before going into such *in vivo* studies, it is of utmost importance to build a strong foundation where developmental–metabolic interactions and their spatio-temporal importance are established. Here, gastruloids offer a good model system where metabolic manipulations, as described below, can easily performed and analysed at the metabolite level, thus building a strong foundation for further *in vivo* studies.

Gastruloids can be grown in large numbers that enable screening for phenotypic consequences of signalling pathway manipulations, or to determine the consequences of teratogens on early stages of development [34]. The same approach can be taken for different metabolic inhibitors or through the manipulation of the physiological environment of the culture medium. For example, it has been demonstrated that Brachyury, one of the earliest markers of mesoderm specification, can be induced in gastruloids by growing them in a hypoxic environment [35]. Taking a high-throughput approach will be critical for the study of metabolism in development as any manipulation has pleiotropic effect on other pathways with the system. A good example is ATP — produced in a handful of reactions and majorly in mitochondria but used in a wide array of metabolic processes. In such cases, it is important to analyse a metabolic phenotype at a metabolome level and requires the ability to generate enough tissue in a reproducible manner. Therefore, caution to be taken not to transplant the experimental logic of developmental genetics to metabolic studies of development. In genetics, a particular perturbation of a given regulatory interaction can be related directly to the observed phenotype. In developmental metabolism, it is critical to interpret the phenotype of a given manipulation, not in terms of the targeted reaction directly, but instead on the systems-level consequence of the manipulation on the metabolome. The ability of gastruloids to then supplement growth media with specific metabolites then enables rescue experiments to test any hypotheses generated from this approach. Such rescue experiments are not possible *in vivo* as there is no direct control over the nutrient supply to the embryo.

Recently, these features of the gastruloid system have been leveraged to show how mannose plays a crucial role in mesoderm specification [36]. The addition of the hexokinase II inhibitor 2-deoxy-d-glucose (2-DG) to gastruloids resulted in an inhibition of mesoderm specification and axial elongation. As a large amount of samples could be obtained, it was possible to study this phenotype at the metabolome level which further confirmed the disruption of glycolysis through a parallel approach of glucose deprivation. Surprisingly, however, this did not inhited mesoderm specification, and the addition of mannose was instead capable of rescuing the 2-DG phenotype. Together, the results point to a critical role for mannose-dependent glycosylation for proper mesoderm specification in gastruloids [36]. This *in vitro* study was complemented by an *in vivo* dataset, also highlighting the role of glycosylation in mesoderm specification during gastrulation [37].

## Variations of gastruloids and alternative models to study metabolism in later embryonic development

As embryonic development proceeds extra-embryonic tissues grow in consort with the embryo to further regulate nutrient availability and metabolism, for example, during chorioalantoic branching stages [20]. Such growing complexity both in the embryonic and the extra-embryonic tissues calls for the need to add additional layers of complexity in gastruloids as well which mimics later stages of post-implantation stages. We have discussed such gastruloid-derived models below and how they can be utilised to study the role of metabolism in the organogenesis stages of the embryonic development.

### Embryoids

Gastruloids are self-organised structures that undergo morphogenesis concomitantly with the generation of signalling gradients to regulate fate specification and patterning [21]. Embryoids are an extension of the basic gastruloid protocol in which an external signalling source is introduced at the time of aggregation [38]. Briefly, embryoids are made by combining two aggregates, one treated with BMP4 and the other kept as naïve ES cells. The BMP4-treated aggregates upon combination act as a signalling centre that induces mesodermal fate within the naïve aggregate [39]. Furthermore, other fates such as ectodermal and endodermal fates are specified. More importantly, mesodermal cells undergo gastrulation-like movements within the aggregates something which gastruloids lack. This system would help us address questions pertaining to the role of metabolism in cell migration during gastrulation. Overall, embryoids exhibit a higher level of tissue organisation and contain structures such as the notochord, gut tube and neural tube. Hence, embryoids offer a good model system to further analyse how metabolism plays an important role in more complex morphogenetic processes other than just the AP elongation in gastruloids.

### Trunk-like structures and *in vitro* somitogenesis

Mesodermal cells are not only formed during gastrulation but continue to be produced from neuro-mesodermal progenitors (NMPs) located in the tailbud of the embryo to contribute to the pre-somitic mesoderm (PSM). In addition, NMPs also form neural cells which subsequently contribute to the spinal cord [40]. Studies as early as 1950 showed that the tailbud is the morphogenetically active region and sensitive to glucose deprivation [41]. Recently, it has been shown how glucose metabolism is important in the PSM development in both the chicken and the mouse [42–44]. Studies conducted in the mouse used the PSM explants derived from the *in vivo* mouse embryo [42,43]. The PSM explant also contains the neural tube and both the neural and the mesodermal cells exhibited the expression of glucose transporters. Neural tube is also an elongating structure like the PSM, and it has been shown that their elongation is interdependent [45]. Any manipulation of glucose metabolism will affect both the structures and making it difficult to ascribe the role of glucose metabolism to either the PSM or the neural tube elongation. Moreover, higher glucose leads to neural tube closure defects highlighting glucose may have an important role to play in the nervous system development as well [46].

Two variants on the gastruloid protocol offer the opportunity to dissociate the impact of glucode metabolism on mesoderm and neural development during body axis elongation [47,48]. Both these structures are formed when gastruloids are embedded in Matrigel, a mixture of extra-cellular matrix proteins, thus adding an extra layer of complexity. This system can be used to address questions pertaining to mechanics and the role of metabolism in that or vice versa in patterning and morphogenesis. Another, way of utilising these structures is to model embryopathies related to somitogenesis, e.g. scoliosis and how metabolism plays an important role in the development of this embryonic disorder. Recent studies using human embryonic stem cells and iPSCs to model somitogenesis *in vitro* can be combined with metabolomics studies not only to understand its implication in human somitogenesis but also congenital disorders as mentioned above [49,50].

### Gastruloids to study heart development

Heart development is a complex patterning and morphogenetic process that starts early in development during gastrulation and continues through the organogenesis stage. At the same time, the embryo starts to switch from the glycolytic mode of metabolism to more oxidative phosphorylation (OXPHOS) [51]. This is also reflected in heart development as well where early cardiomyocytes rely on glucose as an energy source while late stages depend on OXPHOS as mitochondria mature. Any defect in glucose metabolism, especially gestational diabetes, leads to congenital heart defects [52,53], but it is unclear how cells in the developing heart utilise glucose. When gastruloids are treated with a cocktail of three cardiogenic factors — basic fibroblast growth factors, ascorbic acid and vascular endothelial growth factor — on the anterior side of the gastruloid, primary and secondary heart field like progenitors are specified which later organise themselves into cardiac crescent and early heart tube-like beating structure [54]. At the same time, posterior to the heart tube, vascular-like network is also formed. Overall, this *in vitro* structure mimics the embryonic heart development. This would not only help us understand how glucose is used in normal heart development but also to model diabetic cardio-embryopathies simply by changing the glucose concentration in the culture medium or using chemical inhibitors that interfere with glucose metabolism. Such manipulations can be combined with high-throughput microscopy and image analysis, single-cell RNA sequencing and metabolomics to understand the disease mechanism at both cellular and tissue level, gene level and metabolite level, respectively, all of which are technically challenging in the mouse embryo.

### Modelling anterior neural development

In mouse embryos, the regulation of the Wnt pathway is tightly controlled and only the posterior part is responsive to the Wnt activation where the mesoderm is first specified. The anterior epiblast on the other hand inhibits the Wnt pathway to avoid its posteriorisation. Gastruloids, where the Wnt pathway is uniformly activated, represent more the posterior domain of the *in vivo* mouse embryo, and do not have any anterior structure [21]. It has recently been shown when gastruloids were treated with epiblast-inducing factors and a Wnt antagonist to avoid premature specification into mesodermal cells, such gastruloids showed much more enhanced AP patterning including the expression of forebrain/midbrain/hindbrain markers [55]. Not much is known about the role of metabolism in the early anterior neural development. One study on *Xenopus* explants showed that intracellular alkalisation of embryonic cells induces the expression of Otx2, an anterior neural gene [56]. Intracellular pH regulation is very much dependent on the metabolic profile of the cells as it was shown recently in the chicken tailbud and the PSM development [57]. This gastruloid system with the potential of forming anterior neural tissue is a good model to answer questions pertaining to changes in the metabolic profile and its impact on neural fate specification in the mammalian embryo.

### Primordial germ cells and role of metabolism in their differentiation

Primordial germ cells (PGCs) are a specialised type of cells capable of producing gametes and have the capacity to form an organism upon fertilisation. PGCs are specified as early as E7.5, i.e. mid-gastrulation and as the development proceeds, they migrate to the genital ridge and differentiate according to sex [58]. During their development, PGCs undergo various epigenetic changes, and it is known that epigenetic changes are linked to metabolic pathways that provide methyl, acetyl, etc. groups to modify the histone and the DNA, thus regulating gene expression in these cells [59,60]. However, very little is known about the metabolic signature of the PGCs. One study showed that late-stage PGCs display enhanced OXPHOS and reduced glycolysis but how these pathways and their modulation control the early specification remain elusive [61]. The number of PGCs is quite low and for metabolomics analysis, one would need a huge number of embryos to extract PGCs. PG-like cells (PGLCs) can be differentiated in 2D but this lacks spatial tissue organisation as *in vivo*. Gastruloids are a good model to study the early stages of PGC specification as they represent the embryonic gastrulation stages when PGCs are specified. The gastruloid-derived PGLCs also express Blimp1 and Stella which make the epiblast cells commit to the germ cell fate *in vivo* [62]. Additionally, gastruloids also display later stages of germ cell development. There are transgenic lines available to mark the developing PGLCs and as gastruloids can be produced in a high-throughput manner, labelled PGCLs can be isolated from them in high numbers at different stages and subjected to metabolomics analysis.

## Future directions for the field

Metabolic perturbations have largely relied on using the chemical inhibitors which often have pleiotropic effects, i.e. targeting more than one enzyme or the pathway [9]. As metabolic reactions are intricately wired, such pleiotropic effects make it difficult to associate one pathway to a given phenotype. Therefore, developmental metabolism requires the generation of new tools to precisely target individual metabolic enzymes with tight temporal control, and to readout the impact of each perturbation at the systems level.

As the metabolic reactions occur at a very fast pace, strategies need to be implemented that allow rapid and acute targeting of an enzyme to slow or shut down its function. There are multiple systems which would allow targeting enzymes of metabolic pathways individually or in combination [63–65]. The degradation systems include two components — a tag that labels the endogenous protein and the other that recognises this tag and targets the entire fusion protein to degradation. For example, plant hormone Auxin recognises the AID tag added to the genomic locus of a protein and targets it to the proteasomal degradation [63]. Metabolic pathways, once shortlisted using a chemical inhibitor screen, can then be manipulated by conditionally degrading metabolic enzymes. With the advent of genome editing technologies, tagging of endogenous proteins can achieved easily in 2D ES cells. Making gastruloids from such cells provides an initial step towards understanding the role of a particular metabolic pathway in patterning and/or morphogenesis.

Before perturbing a metabolic pathway, it is important to define the metabolic state of a cell type. So far, the metabolic profile of a particular cell type has been described on a population level with the help of bulk metabolomics. This leaves a huge gap in our understanding of the quantitative differences in the reactions of the same metabolic pathway taking place in individual cells, further influencing various cellular function. Another disadvantage of the bulk metabolomics is its discrete nature, i.e. it only highlights stark differences in the metabolic profiles of different cell populations. This is of a concern for studying how one cell type gives rise to another, for example, during cell fate specification. Since in a population of cells, different cells are at different stages of differentiation, studying metabolic changes at a single-cell resolution would provide an opportunity to map the cell differentiation trajectory. Such studies could then be combined with other single-cell technologies to build a metabolism-transcriptional network. The continued development of cellular and sub-cellular methods for probing metabolism will likely lead to important new discoveries in the field of developmental metabolism [66].

As the single-cell metabolomics gives an idea about the quantitative differences in the metabolites’ levels in individual cells, it lacks the spatial information. There are ways to gain spatial information on the metabolic state. For example, bound and unbound forms of NAD and NADH can be imaged using fluorescent lifetime imaging (FLIM) [67]. As various forms of NAD serve as cofactors in various oxidoreductive reactions, their relative fluorescence intensity can used as a readout of a particular metabolic state of cells within a tissue. Recently, it has been shown a posterior–anterior gradient of NAD(P)H in the gastrulating mouse embryo [37]. One limitation is such cofactors are produced and utilised in a wide range of pathways and again this makes it difficult to gain a spatial information on the actual metabolic state of cell *in situ*. This can be overcome with state-of-the-art spatial metabolomics technique, i.e. measuring the metabolite level *in situ.* This technique involves performing mass-spectrometry analysis on a tissue section, often known as the mass-spec imaging technique [68]. One such study showed a tissue-specific abundance of lactate and citrate in a developing mouse embryo [11]. One can also study the flux of carbon atoms *in situ* using heavy isotope-labelled substrates and image them using the technique multi-isotope imaging mass spectrometry (MIMS) [69]. Implementing this technique would provide a more direct and in-depth insight into the metabolic state of a cell with respect to others in a tissue. As gastruloids are self-organising structure, mass-spec imaging technique would allow us to examine how metabolic heterogeneities arise in an otherwise homogenous structure, thus, forming a gradient which further influences the patterning and morphogenesis. This would be solid experimental evidence for Child's metabolic gradient theory in the context of embryonic development.

## Summary

- The field of developmental biology is returning to the study of metabolism as a regulator of developmental processes.
- Gastruloids provide an appropriate assay to uncover mechanisms of metabolic control due to their relative simplicity to the mammalian embryo.
- Variations on the gastruloid protocol enable specific tissues and tissue interactions to be isolated.
- New mechanistic insights from gastruloid studies can then be validated *in vivo* through the design of targeted experiments.

## Open Access

Open access for this article was enabled by the participation of University of Cambridge in an all-inclusive *Read & Publish* agreement with Portland Press and the Biochemical Society under a transformative agreement with JISC.

## Abbreviations

2-DG: 2-deoxy-d-glucose
AP: anterior–posterior
NMPs: neuro-mesodermal progenitor
OXPHOS: oxidative phosphorylation
PGCs: primordial germ cells
PGLCs: PG-like cells
PSM: pre-somitic mesoderm
